# Screening for economic hardship at Child Health Care Centres: A qualitative study of stakeholders’ perceptions and experiences of the Healthier Wealthier Families model in Sweden

**DOI:** 10.1177/14034948241252227

**Published:** 2024-05-30

**Authors:** Nina Johansson, Georgina Warner, Nils Avogadri, Anna Sarkadi

**Affiliations:** Department of Public Health and Caring Sciences, Uppsala University, Sweden

**Keywords:** Poverty, public health, parents, child healthcare, financial counselling

## Abstract

**Aims:**

The Healthier Wealthier Families model uses the child healthcare services as an access point to screen and connect parents experiencing economic hardship to municipal Budget and Debt Counselling services. This study aimed to explore the perceptions and experiences of the Healthier Wealthier Families model in a Swedish context.

**Methods:**

Semi-structured interviews were conducted with three stakeholder groups: eligible parents who declined (*n*=10) and received (*n*=9) financial counselling; nurses (*n*=7); and financial counsellors (*n*=5). The data were analysed using thematic analysis.

**Results:**

The analysis resulted in three main themes conveying the stigma of talking about finance, the connection between economic situation and family wellbeing, and the nuts and bolts of providing preventive financial counselling.

**Conclusions:**

**A working model aiming to ameliorate child poverty in a societal service context needs to address the preconceptions and perceived mandate and role of the professionals, the prevalence of financial stigma in society, especially in relation to being a ‘good’ parent, and the current preoccupations and perceived financial needs and hopes of the families served.**

## Background

Historically, Sweden has developed its social policies towards redistribution and compensation to avoid large income disparities [1]. Unfortunately, investments in family politics and the compensatory effect of financial family policies have diminished over time. Since the end of 1990, the redistributive effect of allowances for families with children has been halved [1], the gaps between socioeconomic groups are increasing, and vulnerable groups are less resilient towards changes in the economy [2].

Increased income inequality and economic hardship can negatively affect parents’ investment in their children [3]. The family stress model refers to the abundant evidence of the negative impact of economic hardship on the emotional home environment, emphasising how stress can affect the parent–child relationship [4]. Family finances create a framework for material possessions, household quality and children’s quality of life [5]. The financial stigma and shame of not being able to uphold that framework can have a strong effect on parental stress. Those affected by economic hardship may doubt if they can be considered a good parent if they cannot provide for their children’s basic needs [6]. This can make it even harder to reach out for help regarding family economy; seeking help may also appear to break a Swedish societal norm of self-sufficiency [6].

In terms of the effects on children, there is growing literature on how children exposed to poverty and economic hardship can experience increased risks of physical and mental health problems [7]; lower social-emotional ability [8]; low levels of school completion [9]; and time outside of the labour market [10]. In addition, economic hardship can also negatively shape children’s image of their future and their role in society [11]. An increase in household cash income in low-income households, on the contrary, is positively associated with improvements in children’s cognitive and behavioural development, school achievement and health [12]. It is, therefore, important to provide support to families with children who are at risk of economic hardship. Examples of support include interventions focusing on monthly cash gifts [13]; neighbourhood-level interventions to improve children’s opportunities [14]; and offering financial counselling in healthcare settings [15].

The Swedish Budget and Debt Counselling (BDC) services are legislated by law [16], to offer free service to people in debt. General guidelines for the service are provided by the Swedish Consumer Agency [17], but the service lacks a national uniform framework, quality criteria and proper evaluations by the responsible inspectorate [18]. Despite the public opinion that the state should provide support for citizens facing economic hardship, the service is not well known publicly [18]. People are usually informed of the service when they have an acute debt situation or risk eviction. However, there has been a push from the governing agency for financial counsellors to provide more preventive services [18].

Swedish Child Health Care (CHC) is a publicly financed and universal service [19], having a broad coverage of the Swedish population [20]. Nurses at CHC build relationships with families through the frequent visits during the child’s first year of life, and annual visits thereafter. They are in a position to detect psychosocial risk factors in young families, and function as a bridge to available societal services [21]. However, there is a general lack of comfort in performing screening and addressing topics such as financial matters among clinicians [22], although using structured methods alleviates approaching the subject [21]. Moreover, previous studies have described difficulties in implementing universal screening of social determinants of health in healthcare settings [23]. Striving for efficient clinical flow and clinicians’ perceptions of need among patients have been identified as potential obstacles to screening. Yet, there are examples demonstrating that identifying families experiencing, or at the risk of, economic hardship, and linking them to available support can be a way forward [23]. One such example is the model Healthier Wealthier Families (HWF) [15].

**Figure 1.**
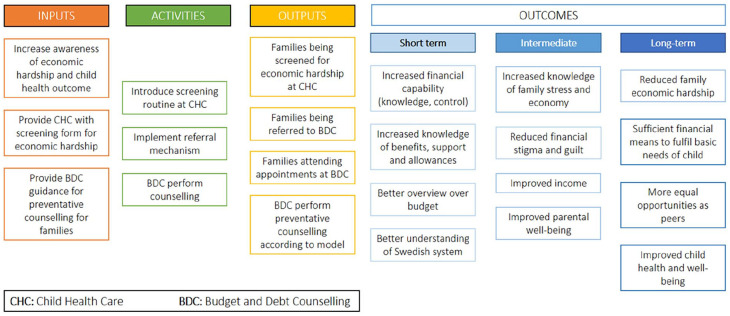
Healthier Wealthier Families logic model.

The HWF model aims to identify parents in economic hardship through the CHC service, and create a referral pathway to the BDC services. Model components and anticipated outcomes are described in the logic model (Figure 1). HWF originates from Scotland, under the name Healthier Wealthier Children [24], and was imported to Sweden through international collaboration by our research team [15].

The HWF model was adapted to the Swedish context in a setting of a municipality with 39,290 habitants in mid-Sweden [25], with a relative child poverty level (income under 60% of the national median) of 27% [26], one main CHC centre, and a BDC service contracted from a neighbouring municipality. HWF was introduced to a municipality in Sweden concurrently with the commencement of this evaluation. The evaluation aimed to explore stakeholders’ perceptions and experiences of the HWF model in Sweden.

## Methods

### Design

The study had an exploratory design, with qualitative semi-structured interviews.

### Participants and sampling

The local CHC and BDC service were invited to the study, and all stakeholders accepted. The professional study sample comprised nurses at CHC (*n*=7) and financial counsellors (*n*=5). The parents were selected using three inclusion criteria: being a parent to at least one child aged 0–5 years; enlisted at the participating CHC; and at least one self-reported risk factor for economic hardship (see Table I for screening questions). The study sample consisted of both parents who were eligible, but declined BDC (*n*=10), and parents who received the service (*n*=9).

**Table I.** Screening questions for economic hardship.

Screening question
During the last 12 months, have you been worried that your family will run out of money at the end of the month?
During the last 12 months, have you been able to pay current expenses, such as rent, bills, insurance?
During the last 12 months, have you had the opportunity to buy necessary items, such as clothes and food for yourself and your children?
Is there at least one person in your household who currently has a paid job?
Would you be able to handle an unexpected expense of 20,000 SEK (approx. £1500) without asking for help or borrowing money?

### Data collection

The researchers developed interview guides for the three stakeholder groups, guided by the model of process evaluation for public health interventions and research of Steckler and Linnan [27]. Each process component was considered, in which for example *context* guided the questions on if the CHC service was the right arena for screening for economic hardship, *reach* was the basis for the questions on whether or not they chose to participate, and *dose* informed the questions if the participants received what was promised on enrolment [27]. See supplemental attachments for the interview guides.

The semi-structured interviews were conducted through telephone and a video teleconference platform, from April to September 2021, by the first author (NJ), third author (NA), and a research assistant. The interviews were recorded digitally, transcribed verbatim, and lasted between 15 and 60 minutes. Seven parental interviews were conducted with interpreters in Arabic and Tigrinya. A quality check of the translations was conducted for a sample of the interview transcripts.

### Data analysis

The analysis process was iterative, inductive and guided by the six phases of reflexive thematic analysis [28]. The first author (NJ) and third author (NA) commenced the process of: (a) familiarising themselves with the material; followed by (b) generating initial codes; and (c) searching for themes. At stage (d) of reviewing the themes and (e) defining and naming the themes, the second author (GW) and last author (AS) joined the process. NJ took the lead in (f) writing up the material, with support from all authors. All authors are, or have been, affiliated to the same research team. NJ is a PhD student, GW is an associate professor and AS is a professor and team leader. NA is a former medical student affiliated to the team for his master’s thesis. No qualitative analysis software programme was used in the process.

### Ethics

The study was conducted according to the ethical research guidelines of the Swedish Ethical Review Authority and received ethical approval in January 2021 (Dnr 2020-06738). Informed consent was documented for all participants.

## Results

The analysis resulted in three main themes with sub-themes (Table II). In the following section, the themes are presented together with quotes.

**Table II.** Themes and subthemes.

Main categories	Sub-categories
‘Talking about finances is so taboo!’ Or is it?	The anticipated obstacle of the topic
	The personal relationship – to cherish and to build on
	The norm and the façade – friend or foe
Financial wellbeing is family wellbeing	How to work a wicked problem
	Being a parent in society today
	Economic hardship and the child focus
The how, who and why of working preventively	Who is it really for?
	Constraints of time and resources
	Experiencing financial counselling
	Expectations and conditions – to lead or to get in the way

### Theme 1: Talking about finances is so taboo! Or is it?

The sub-theme ‘The anticipated obstacle of the topic’ describes how discussing finances is strongly associated with taboo and stigma, but also how this feeling might be a projected perception. The nurses expressed an anticipation that talking about finances can upset parents, regardless of their financial situation. The financial counsellors agreed with this view. However, the parents expressed no major discomfort in discussing the topic with the nurses. The group acknowledged the taboo nature of the topic, highlighting that finance is not a subject they normally would bring up with anyone. However, they also stressed the importance of overcoming financial taboo, due to finances being such a natural part of the everyday family life.

Since we have to talk about finances, we have a way in. But generally, it can be difficult to bring it up. (Counsellor 4)

Well, no, I have no taboo in discussing my finances [laughter]. (Parent 1)

The sub-theme ‘The personal relationship – to cherish and to build on’ refers to the emphasis on a personal relationship when handling a difficult subject. All nurses emphasised that they want to build good relationships with their families. Parents agreed and gave examples of occasions they had felt important in the eyes of the nurses. Both groups talked about how an established relationship can alleviate bringing up sensitive topics, which, in turn, can further strengthen the relationship. Several financial counsellors vouched for this as well, and underlined the pandemic disruption as an obstacle for the in-person-meetings.

We as nurses want to build relationships with our families. And it gave further depth to our relationships to include these questions about finances. (Nurse 5)

Even though she [the nurse] meets many families she always makes us feel remembered. That she knows who we are. (Parent 2)

The sub-theme ‘The norm and the façade – friend or foe’ indicates a strong norm of what economic hardship looks like and implies. Many parents described a strong distinction between having sound financial practices yet experiencing a hard time, and having economic hardship due to reckless behaviour. Several professionals, both nurses and financial counsellors, talked about the risk of performing ‘pre-screenings’, that is, deciding on which families to screen beforehand on the basis of their own preconceptions.

Some parents have all external attributes that show ‘we’ve got money’. To then screen them feels bad. (Nurse 2)

No mother on social media shows the dull days with clothes covered in vomit or buying the cheapest wipes. For a first-time parent, that’s hard. (Parent 3)

### Theme 2: Financial wellbeing is family wellbeing

The sub-theme ‘How to work a wicked problem’ was built on the perception of economic hardship being a multifaceted or ‘wicked’ problem. An example of the ‘wickedness’ was the financial counsellors’ reflection that mental health problems can be the starting point of economic hardship, but also that long-term economic hardship can cause mental health problems. Parents highlighted that facing personal problems while handling the Swedish bureaucracy and managing finances is a challenge during the first years. Both the nurses and financial counsellors agreed that to handle economic hardship, there is a need for joint societal interventions.

It’s important that families get help quickly, after identified hardship. If it takes too long, we might lose them. (Nurse 6)

I think it saves money and suffering for children, the family and the society. You certainly shouldn’t underestimate the cost of an unbalanced economy in terms of mental health, sickness absence rates, etc. (Counsellor 5)

The sub-theme ‘Being a parent in society today’ highlights the experience of parenthood in a difficult societal structure and culture. Parents stated that the financial perspective is a common source of stress, in which it can either be something existing or something that develops over time. A significant share of the three stakeholder groups testified that being a parent in Sweden today involves the challenge of fully grasping the societal system of insurances and transferences related to childrearing.

It takes preparation. Otherwise, they stand there with their child like ‘oh, did I get this low parental benefit?’ (Counsellor 4)

I moved to a new city, enrolled my kids into preschool. Later, I was fined 1800 SEK. Authorities claimed I did something wrong with the paperwork. I tried to talk to them, they were mistaken. It is difficult, not speaking Swedish. (Parent 1, declined BDC)

The sub-theme ‘Economic hardship and the child focus’ describes how children can be negatively affected by economic hardship. Parents, nurses and financial counsellors all talked about the parental worry of being unable to provide for the family, beyond bare necessities. The groups talked about the different expenses that the parental community perceive as ‘normal’ or expected, such as throwing a birthday party. They agreed that parents make sacrifices for their children, to live up to internal and external expectations.

We see that finances are a very important factor, and that poor finances affect child health a lot. (Nurse 2)

We were invited to a birthday party. Luckily, we had gotten the child allowance. When you attend a party, you don’t want to be the child without a gift. (Parent 6)

### Theme 3: The how, who, and why of working preventively

The sub-theme ‘Who is it really for?’ highlights the discussion regarding who the BDC services are there for. The financial counsellors talked on one hand about the goal of being a universal service, and on the other hand about which clients can benefit from counselling. Some financial counsellors expressed difficulties in having to prioritise someone with lighter problems, a more preventive counselling, over someone with long-term debts. Parents’ sentiments aligned: some parents recalled having ‘too light’ problems to receive ‘proper’ counselling, while others mentioned having ‘too difficult’ situations to receive counselling.

If someone with debts is granted a reconstruction, I have a result of my input. But to measure preventive work, what service have I provided? Did I help? (Counsellor 5)

My financial situation didn’t allow full financial counselling. My expectation was to learn about family life, finances. I wanted to learn, but my situation was too bad so, I couldn’t do anything about it. (Parent 7)

The sub-theme ‘Constraints of time and resources’ refers to capacity issues within each service. Both nurses and financial counsellors talked about having a high workload and time constraints. This was seen in statements of ways to save time, like pre-screening. Within both services, there was a wish to develop a preventive way of working together. However, nurses and financial counsellors emphasised that the routine way of working might need to change for that to happen. Among parents, time was described as an obstacle to take on new tasks and start working with their finances.

I needed more time. You had to have a good way in with a family. (Nurse 5)

The main reason we say no to counselling is that we don’t have the time. But of course, that changes as the children get older. (Parent 2, declined BDC)

The sub-theme ‘Experiencing financial counselling’ explains how the BDC, in general, is an unknown service. The nurses talked about not having prior knowledge of the service, but how they appreciated the new knowledge of where to refer to, when needed. Financial counsellors were aware of their status and expressed a wish to reach out more. At the same time, there was a fear of the service not having enough capacity to meet potential demand. For parents, counselling provided an opportunity to share the burden of economic hardship and create concrete action plans.

If I ask questions [about finances], I need to be able to handle the answers. To guide them to get help. (Nurse 7)

Prevention is something we always talk about. But we mostly just keep working. In order not to have such a long queue. (Counsellor 3)

The sub-theme *‘*Expectations and conditions – to lead the way or to get in the way’ describes how different expectations shaped the stakeholders’ views of their participation. Parents who had an over-optimistic view of the capacity of the BDC service, shaped from TV shows and social media, were disappointed with the service. Being largely unknown, this was highlighted as a common problem among the financial counsellors, not living up to these expectations. The financial counsellors who expressed a low personal interest in prioritising clients with no obvious acute problems, also expressed low levels of engagement in changing the way they work.

Sometimes clients are optimistic of what a counsellor can do. That we have more power, as seen on TV [the Swedish version of Luxury Trap]. (Counsellor 4)

I really saw this as a great opportunity to offer something to the families. That it could be a great help. (Nurse 1)

## Discussion

The aim of this study was to explore the perceptions and experiences of the HWF model in a Swedish context. Our findings showed that stakeholders generally felt that economic hardship in families is an important subject, and that more work needs to be done. Yet, the findings showed internal and external obstacles in adapting to a new working model.

Similar to previous research, we detected obstacles in implementing the model [6, 15, 18, 19, 23]. Obstacles included professionals experiencing a high work load and needing to make priority-based decisions [18, 23], the prevalence of financial stigma [6], the ambiguities regarding professional roles and the service mission [19], and the impacts of the COVID-19 pandemic [15].

Similar to findings from previous HWF research [15], this study found no statements of major parental discomfort in discussing financial matters in the realms of a trusting relationship. The parents did, however, acknowledge the potential stigma around financial difficulties. Nurses, in turn, were apprehensive and anticipated the subject being taboo. The latter aligns with preceding literature of healthcare professionals expressing discomfort with addressing matters of finances [22], together with the knowledge that stigma and economic hardship are related [6]. Our findings can therefore be discussed in terms of professionals’ discomfort, together with an anticipated stigma related to financial problems within their clientele. This might have led to the ‘pre-screening’ practices described by the nurses.

To a large extent, the results of this research support previous literature on addressing adverse childhood experiences, and the challenges of integrating screening processes on what can be considered sensitive topics [15, 22, 23, 29]. Engström et al. reached similar conclusions in their qualitative study on screening for psychosocial risk [29], in which nurses emphasised the importance of sufficient time and the foundation of a trusting relationship to work with these risk factors. Our findings align with previous research in which nurses in their roles as well-respected professionals, could lead a paradigm shift in the views of health issues related to poverty [30]. In our results, we also found a view of financial counsellors as a promising component to reduce social and material deprivation and to alleviate financial stress, similar to the study of the HWF model in Australia [15]. In fact, Dubowitz et al. [23] highlighted the importance of only screening for risk factors when there are established referral processes and available services.

We were intrigued to see how the execution of the HWF model deviated from the professionals’ perceptions of the importance of addressing financial issues. Professionals perceived a need, both from recent reports [1, 11], and in experiences from the routine service [29], wanting to make a change but then facing a number of direct and indirect obstacles. When describing the obstacles among professionals, it all boiled down to expectations: what is expected of their own working role in relation to resources and responsibilities? This coincides with the Australian HWF study, in which participating nurses were challenged in their perception of their role and mission [15].

Among parents, relationships and autonomy were emphasised: relationships within and close to the family, and autonomy in the form of wanting to solve private matters within the family, to function within the Swedish system and to make use of available support. Although most interviewed parents did not mind being asked about finances within the child health service setting, the sense of potential stigma was very present and appearances were important. A large proportion of the parents described a distinction between having sound financial practices and reckless financial behaviour as a cause of financial distress. With the findings from previous literature [6, 23, 30] and our recent study, we could see a risk of this mindset adding to the aspects of shame in admitting to economic hardship and seeking help.

## Conclusions

Economic hardship can impact family health and wellbeing. However, it is a complex issue to address through societal services. A working model aiming to ameliorate child poverty in a societal service context needs to address the preconceptions and perceived mandate and role of the professionals, the prevalence of financial stigma in society, especially in relation to being a ‘good’ parent, and the current preoccupations and perceived financial needs and hopes of the families served. The perceptions and experiences of HWF in Sweden provide important insights for future implementation and evaluation of the model.

## Supplemental Material

sj-docx-1-sjp-10.1177_14034948241252227 – Supplemental material for Screening for economic hardship at Child Health Care Centres: A qualitative study of stakeholders’ perceptions and experiences of the Healthier Wealthier Families model in Sweden Supplemental material, sj-docx-1-sjp-10.1177_14034948241252227 for Screening for economic hardship at Child Health Care Centres: A qualitative study of stakeholders’ perceptions and experiences of the Healthier Wealthier Families model in Sweden by Nina Johansson, Georgina Warner, Nils Avogadri and Anna Sarkadi in Scandinavian Journal of Public Health
