# Supplementary material for: Screening for economic hardship at Child Health Care Centres: A qualitative study of stakeholders’ perceptions and experiences of the Healthier Wealthier Families model in Sweden
Source: Scand J Public Health. 2024 May 30;53(4):406–12. doi: 10.1177/14034948241252227 (PMC12048729; doi:10.1177/14034948241252227)
Supplement: sj-docx-1-sjp-10.1177_14034948241252227 – Supplemental material for Screening for economic hardship at Child Health Care Centres: A qualitative study of stakeholders’ perceptions and experiences of the Healthier Wealthier Families model in Sweden [file sj-docx-1-sjp-10.1177_14034948241252227.docx]

**Healthier Wealthier Families (HWF), Interview guides**

**Families who received BDC**

| 1. What do you think about that BVC-nurses ask questions about families’ economic situation?  *How did you feel about being asked about you’re the economy of your family at the BVC-visit?* |
| --- |
| 1. What do you associate with the term “economic difficulties”? |
| 1. Have you heard about economic counselling before?  *Is it a service that you have used?* |
| 1. What motivated your participation in the HWF-project? |
| 1. What where your expectations of the HWF-project? |
| 1. As a participant in the HWF-project, do you feel that you have received the things that was promised at the time for enrolment? *If not, what have been missing?* |
| 1. If you were to give any advice on how to improve this project in the future, what would that be? |
| 1. Have your view of economy changed after you participation in the HWF project? |
| 1. After participating in the HWF-project, do you feel equipped to continue working with your family economy?  *What could be obstacles? What could be facilitators?* |
| 1. Would you recommend financial counselling to others, and why? |

# Families who did not receive BDC

| 1. What do you think about that BVC-nurses ask questions about families’ economic situation? |
| --- |
| 1. How did you feel about being asked about your economic situation at the BVC-visit? |
| 1. What do you think about when you hear the term “economic difficulties”? |
| 1. You did not to take part of the HWF, what was the main reason for not participating? |
| 1. What would make you use the service of financial counselling provided by the municipality? |

**Participating financial counsellors**

| 1. How do you feel about the approach to work preventive towards families with children, in collaboration with the health care sector? |
| --- |
| 1. Do you think it is an approach that is suitable for your working arena?  *Is it different from how you usually work? How?* |
| 1. What were your expectations on the HWF project? |
| 1. Has the project lived up to your expectations? |
| 1. As a financial counsellor in the HWF project, do you feel that you have been given the working conditions that was promised at the time for project start *If not, what have been missing?* |
| 1. What have facilitated/created obstacles your work with the project? |
| 1. How have you experienced the collaboration between your organisation and the health care sector within the project? |
| 1. After participating in the HWF project, is this way of working something you would like to maintain and implement?  *In what way?* |
| 1. Would you recommend this way of working, with a focus on interprofessional collaboration and prevention, to others within your field, and why? |
| 1. If you were to give any recommendations on further development of the HWF project, what would that be? |

**Participating CHC nurses**

| 1. Do you consider BVC being the right arena for talking about economy? |
| --- |
| 1. How have you felt about talking to visiting families about their family economy? *Was your opinion different when you started this project?* |
| 1. How did families tend to react when you asked them about their family economy? |
| 1. When being asked to participate in the HWF project, how did the families motivate their willingness or unwillingness to participate? |
| 1. What where your expectations on the HWF project? |
| 1. As a child health nurse within the HWF project, do you feel that you have been given the proper working conditions that was promised at the time for project start? *If not, what have been missing?* |
| 1. Are you happy with the collaboration between your organisation and the financial counsellors within the HWF project? |
| 1. What have facilitated/been an obstacle your work with the project? |
| 1. After participating in the HWF project, do you feel equipped to continue working with visiting families and their economy?  *If not, why?* |
| 1. Would you recommend this way of working, with a focus on collaboration and prevention, to others within your field, and why? |
| 1. If you were to give any recommendations on further development of the HWF project, what would that be? |
